# Protective and therapeutic role of 2-carba-cyclic phosphatidic acid in demyelinating disease

**DOI:** 10.1186/s12974-017-0923-5

**Published:** 2017-07-21

**Authors:** Shinji Yamamoto, Kota Yamashina, Masaki Ishikawa, Mari Gotoh, Sosuke Yagishita, Kensuke Iwasa, Kei Maruyama, Kimiko Murakami-Murofushi, Keisuke Yoshikawa

**Affiliations:** 1 Department of Pharmacology, Faculty of Medicine, Saitama Medical University, 38 Moro-hongo, Moroyama-machi, Iruma-gun, Saitama, 350-0495 Japan; 2 Endowed Research Division of Human Welfare Sciences, Ochanomizu University, 2-1-1 Ohtsuka, Bunkyo-ku, Tokyo, 112-8610 Japan

**Keywords:** Cuprizone, Demyelination, Experimental autoimmune encephalomyelitis (EAE), Microglia, Multiple sclerosis, Neuroinflammation

## Abstract

**Background:**

Multiple sclerosis is a neuroinflammatory demyelinating and neurodegenerative disease of the central nervous system characterized by recurrent and progressive demyelination/remyelination cycles, neuroinflammation, oligodendrocyte loss, demyelination, and axonal degeneration. Cyclic phosphatidic acid (cPA) is a natural phospholipid mediator with a unique cyclic phosphate ring structure at the *sn*-2 and *sn-*3 positions of the glycerol backbone. We reported earlier that cPA elicits a neurotrophin-like action and protects hippocampal neurons from ischemia-induced delayed neuronal death. We designed, chemically synthesized, and metabolically stabilized derivatives of cPA: 2-carba-cPA (2ccPA), a synthesized compound in which one of the phosphate oxygen molecules is replaced with a methylene group at the *sn-*2 position. In the present study, we investigated whether 2ccPA exerts protective effects in oligodendrocytes and suppresses pathology in the two most common mouse models of multiple sclerosis.

**Methods:**

To evaluate whether 2ccPA has potential beneficial effects on the pathology of multiple sclerosis, we investigated the effects of 2ccPA on oligodendrocyte cell death in vitro and administrated 2ccPA to mouse models of experimental autoimmune encephalomyelitis (EAE) and cuprizone-induced demyelination.

**Results:**

We demonstrated that 2ccPA suppressed the CoCl_2_-induced increase in the Bax/Bcl-2 protein expression ratio and phosphorylation levels of p38MAPK and JNK protein. 2ccPA treatment reduced cuprizone-induced demyelination, microglial activation, NLRP3 inflammasome, and motor dysfunction. Furthermore, 2ccPA treatment reduced autoreactive T cells and macrophages, spinal cord injury, and pathological scores in EAE, the autoimmune multiple sclerosis mouse model.

**Conclusions:**

We demonstrated that 2ccPA protected oligodendrocytes via suppression of the mitochondrial apoptosis pathway. Also, we found beneficial effects of 2ccPA in the multiperiod of cuprizone-induced demyelination and the pathology of EAE. These data indicate that 2ccPA may be a promising compound for the development of new drugs to treat demyelinating disease and ameliorate the symptoms of multiple sclerosis.

## Background

Multiple sclerosis is an inflammatory demyelinating and neurodegenerative disease of the CNS characterized by recurrent and progressive demyelination/remyelination cycles, neuroinflammation, oligodendrocyte loss, demyelination, and axonal degeneration [1–3]. In multiple sclerosis, oligodendrocytes are the target of inflammatory attacks and their cell death mediated by activated lymphocytes, macrophages, and glial activation results in axonal demyelination. Since myelin-forming oligodendrocytes provide critical support to the neuronal axon, demyelination results in diverse neurological symptoms determined by the functions of affected neurons. Therefore, therapies designed to protect oligodendrocytes and myelin during neuroinflammation are important strategies to halt the progression of multiple sclerosis.

The most commonly studied animal models of multiple sclerosis are the autoimmune experimental autoimmune encephalomyelitis (EAE) model [4, 5] and the cuprizone (bis-cyclohexanone-oxalyldihydrazone, CPZ)-induced demyelination model [6, 7]. Extensive research regarding the detailed mechanisms underlying immune-mediated demyelination in multiple sclerosis has been conducted using EAE model mice [4, 5]. Activated autoreactive T cells proliferate and release cytokines, which disrupt the blood-brain barrier, and secrete chemokines that lead to the recruitment of T cells, B cells, and macrophages. Infiltrated immune cells secrete autoantibodies against the myelin sheath, ultimately resulting in myelin degeneration [8]. The cuprizone model is characterized by the apoptotic death of mature oligodendrocytes [7] and is accompanied by neuroinflammation and motor dysfunction [9]. The model is used to study processes of demyelination and remyelination in the CNS. Primary oligodendrocyte apoptosis in connection with microglial activation are the major histopathological hallmarks of the cuprizone animal model. These pathological features are also characteristics of lesion formation in human multiple sclerosis [10]. Mitochondrial dysfunction is also an important component of human multiple sclerosis lesions and plays a key role in the loss of oligodendrocytes and axons, which can be observed in both the EAE and cuprizone models.

Cyclic phosphatidic acid (cPA) is a natural phospholipid mediator with a unique cyclic phosphate ring structure at the *sn*-2 and *sn*-3 positions of the glycerol backbone. cPA elicits a neurotrophin-like action [11] and protects neurons from mitochondrial dysfunction-induced apoptosis [12] and ischemia-induced delayed neuronal death [13]. We have also reported previously that cPA suppresses cuprizone-induced demyelination and motor dysfunction [14]. We designed, chemically synthesized, and metabolically stabilized derivatives of cPA: 2-carba-cPA (2ccPA), a synthesized compound in which one of the phosphate oxygen molecules is replaced with a methylene group at the *sn-*2 position; this showed much more potent biological activity than natural cPA [15–17]. Our preliminary experiments revealed that 2ccPA was detected in the mouse brain following intraperitoneal administration. Based on this finding, we speculated that circulated 2ccPA may gain access to the brain via the blood-brain barrier. Currently, we are investigating the pharmacokinetics of 2ccPA in a separate study.

In the present study, we investigated whether 2ccPA exerts protective effects in oligodendrocytes and suppresses pathology in EAE and cuprizone-induced mouse models of multiple sclerosis. We demonstrated that 2ccPA protected oligodendrocytes via suppression of the mitochondrial apoptosis pathway, suppressed cuprizone-induced demyelination and motor dysfunction, and attenuated the clinical symptoms of EAE.

## Methods

### Pharmacologic agents

2-Carba-cPA (2ccPA) was chemically synthesized as previously described (Fig. 2a) [16, 18]. For in vivo experiments, 2ccPA was dissolved in saline (vehicle). For in vitro experiments, 2ccPA was dissolved in phosphatase-buffered saline (PBS) containing 0.1% fatty acid-free bovine serum albumin (BSA) (vehicle).

### Cell culture and treatments

The MO3.13 cell line (CELLutions Biosystems, Inc.) is an immortalized human-human hybrid line that expresses the phenotypic characteristics of primary oligodendrocyte [19, 20]. MO3.13 cells were cultured in Dulbecco’s Modified Eagle Medium (DMEM) (Nacalai Tesque) supplemented with 10% fetal bovine serum (FBS, Gibco), penicillin, and streptomycin (Gibco) in a humidified 5% CO_2_ incubator at 37 °C. MO3.13 cells attain a flattened bipolar morphology with elongated processes and can be differentiated into an oligodendrocyte phenotype. To induce differentiation, MO3.13 cells were cultured in DMEM without FBS for 5 days. Serum-starved differentiated MO3.13 cells were exposed to CoCl_2_ (500 μm) and/or 2ccPA (10 μm) for 2 days. MTT (5 mg/ml) was added to each well, followed by incubation at 37 °C for 4 h in a CO_2_ incubator. The supernatants were carefully removed, and 200 μl of isopropanol was added to each well. The optical density (OD) of the solution was measured at 570 nm using a microplate reader (BIO-RAD).

### Western blotting analysis

MO3.13 cells were harvested with ice-cold PBS, homogenized in ice-cold RIPA (50 mM Tris-HCl pH 8.0, 150 mM NaCl, 5 mM ethylenediaminetetraacetic acid (EDTA), 1% NP-40, 0.1% SDS, 0.5% deoxycholate) containing a protease inhibitor cocktail (Roche) and left at 4 °C for 30 min. The homogenates were centrifuged at ×20,000*g* at 4 °C for 15 min, and the resulting supernatants were collected as whole-cell lysates, from which protein concentrations were determined using a protein assay kit (Thermo Scientific). Proteins were separated on conventional 12% acrylamide SDS gels and transferred to nitrocellulose membranes. After blocking with 5% skim milk (MEGMILK SNOW BRAND Co. Ltd.) in PBS containing 0.05% Tween 20 (PBS-T), the membranes were incubated with the appropriate primary antibodies (anti-GAPDH (Millipore, 1:1000), anti-Bax (cell signaling, 1:1000), anti-Bcl-2 (cell signaling, 1:1000), anti-phospho-JNK (p-JNK, Cell Signaling, 1:1000), anti-phospho-p38MAPK (p-p38MAPK, cell signaling, 1:1000), anti-myelin basic protein (MBP, Santa cruz, 1:1000), anti-oligodendrocyte transcription factor 1 (Olig1, Rockland, 1:1000), anti-glial fibrillary acidic protein (GFAP, Epitomics, 1:1000), anti-glutamine synthetase (GS, Abcam, 1:1000)) overnight, followed by incubation with horseradish peroxidase-conjugated secondary antibodies for 2 h at room temperature. After washing with PBS-T three times, the membranes were treated with reagent for exposure (Chemi-Lumi One Super, Nacalai Tesque; ImmunoStar LD, Wako). Images of the membranes were captured using a C-DiGit Blot Scanner (LI-COR) and subjected to ImageJ 1.46r analysis.

### Animal procedures

Mice were housed in appropriate animal care facilities at Saitama Medical University (Saitama, Japan) and handled in accordance with established international guidelines. Experimental protocols were approved by the Animal Research Committee of Saitama Medical University. C57BL/6J mice (Tokyo Laboratory Animals Science) were received at our facility at 10 weeks of age. Mice were maintained on a 12/12-h light/dark cycle. For histology, mice were intracardially perfused with 4% paraformaldehyde (PFA) in PBS. The brain and lumbar spinal cord tissues were removed and post-fixed overnight in 4% PFA in PBS, following which they were cryoprotected in 30% sucrose solution in PBS, snap frozen, and stored at −80 °C until further use. Coronal brain sections (25 μm) were obtained using a cryostat (CM1900, LEICA) and mounted on gelatin-coated glass slides [21].

### Induction of cuprizone model and 2ccPA treatment

**Fig. 1:**
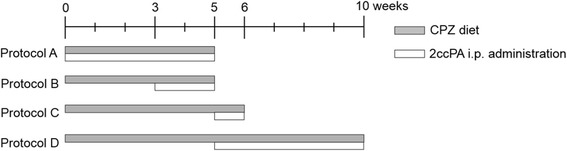
Multiperiod of 2ccPA administration protocol. For protocol A, 2ccPA was administered once daily via intraperitoneal injection for the duration of the 5-week period of cuprizone exposure (0–5 weeks: acute peak demyelination). For protocol B, 2ccPA was administered once daily via intraperitoneal injection between weeks 3–5 of cuprizone exposure (3–5 weeks: after onset of demyelination). For protocol C, 2ccPA was administered once daily via intraperitoneal injection between weeks 5–6 of cuprizone exposure (5–6 weeks: spontaneous remyelination). For protocol D, 2ccPA was administered once daily via intraperitoneal injection between weeks 5–10 of cuprizone exposure (5–10 weeks: chronic demyelination)

**Fig. 2:**
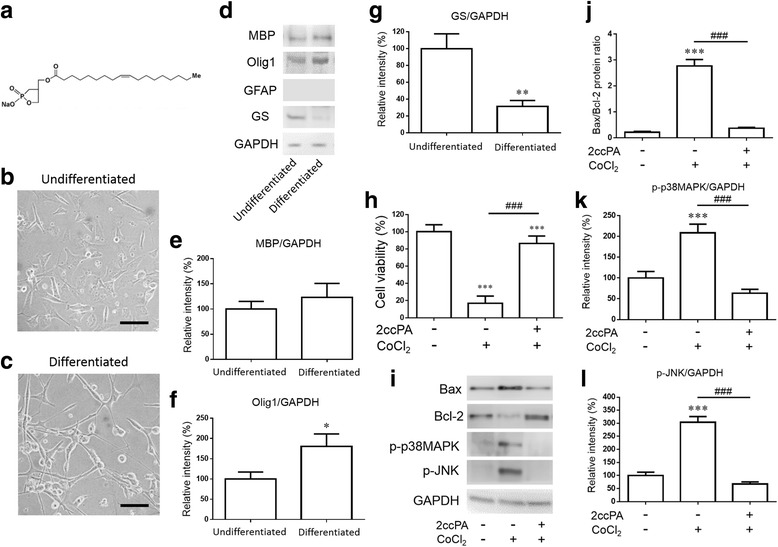
Structure and effect of 2ccPA on CoCl_2_-induced apoptosis. Structure of 2ccPA 18:1. 2ccPA is the compound in which one of the phosphate oxygen is replaced with a methylene group at the *sn*-2 position (**a**). Chemically synthesized cPA derivative, 2ccPA, used for the present experiments. Light microscopic undifferentiated MO3.13 cells (**b**) and differentiated MO3.13 cells (**c**). *Scale bar* = 100 μm. The protein levels of MBP, Olig1, GFAP, GS, and GAPDH determined by Western blot analysis (**d**). Protein levels of MBP (**e**), Olig1 (**f**), and GS (**g**). Data are presented as the mean ± SEM, *n* = 6. Statistical analysis were performed using a student’s *t* test. (*p < 0.05; **p < 0.01 vs. control). MO3.13 cells were incubated with 500 μm CoCl_2_ in the presence of 10 μm 2ccPA. Cell viability of MO3.13 cells were evaluated using MTT assay (**h**). The protein levels of Bax, Bcl-2, phosphorylated-p38MAPK (p-p38MAPK), phosphorylated-JNK (p-JNK), and GAPDH were determined by Western blot analysis (**i**). The expression of Bax and Bcl-2 was determined using a densitometer, and the Bax/Bcl-2 ratio was calculated (**j**). Protein levels of p-p38MAPK (**k**) and p-JNK (**l**). Data are mean ± SEM, *n* = 6. Statistical analysis was performed using one-way ANOVA followed by post hoc Newman-Keuls test (***p < 0.001 vs. control; ^###^ *p* < 0.001 vs. CoCl_2_)

Male C57BL/6J mice were given ad libitum access to a powdered diet (CLEA Japan) containing 0.2% bis-cyclohexanone-oxaldihydrazone (cuprizone, Merck KGaA). Mice were fed the cuprizone diet for 5 weeks (acute peak demyelination), 6 weeks (spontaneous remyelination), and 10 weeks (chronic demyelination). In the present study, 2ccPA was chemically synthesized as previously described (Fig. 2a) [17], dissolved in saline, and administered at a dose of 1.6 mg/kg via intraperitoneal injection once daily during the cuprizone exposure period (0–5, 3–5, 5–6, or 5–10 weeks from the onset of exposure). The protocol for 2ccPA administration is presented in Fig. 1. Control mice were fed a cuprizone-free diet and received an equal dose of saline via intraperitoneal injection once daily during each experimental period. Coronal brain sections were stained for myelin using Black-Gold II (Histo-Chem) as previously described [22]. Briefly, sections were incubated in a 0.3% Black-Gold II solution for 12 min, rinsed in distilled water, fixed in 1% sodium thiosulfate, rinsed in tap water, and air-dried. Sections were coverslipped using Poly-Mount (Polysciences Inc). Black-Gold II (Histo-Chem) stained sections were selected between Bregma −0.22 and −0.58 mm. Sections were photographed at ×10 magnification on a KEYENCE BZ-X700 microscope (Keyence Corporation). Images were captured using a KEYENCE BZ-X700 BZ-X Analyzer and imported into ImageJ 1.46r, which was used to measure the mean OD within the middle of the corpus callosum [21]. The OD of the tissue-free area was used as a background, and blank was subtracted from the ODs for tissue. The resulting ODs for myelin in each mouse were normalized against values in unchallenged mice using the following formula: myelin score (%) = (density reading/unchallenged density average) × 100.

### Electron microscopy analysis of myelin sheath thickness and axon diameter in the cuprizone model

Mice were anesthetized and perfused with PBS as described in the preceding sections, following which they were fixed using 4% PFA and 2.5% glutaraldehyde in phosphate buffer and re-fixed overnight at 4 °C. The fixed brains were sliced into 1–2-mm sections. Sections containing the corpus callosum area were divided into segments of 2–3 mm and washed in 0.1 M sodium cacodylate buffer. After washing, the tissue was post-fixed with 1% osmium tetroxide. Sections were dehydrated in an ascending alcohol series and embedded in epoxy resin. Ultrathin sections of the corpus callosum were prepared using a Reichert-Nissei ULTRACUT-N ultramicrotome (Nissei Sangyo). Ultrathin sections were stained with lead nitrate and 3% uranyl acetate in water. Digital images were acquired using a JEM-1400 Transmission Electron Microscope (JEOL Ltd.). Ultrathin sections (70 nm) were examined with a transmission electron microscope at 80 kV [23]. The number of myelinated fibers present in electron microscope pictures was counted. Using ImageJ 1.46r, axonal diameters were calculated from the outer perimeter of the axon divided by the total perimeter of the axon. The g-ratio were calculated the numerical ratio between the diameter of the axon proper and the outer diameter of the myelinated fibers. Pictures were randomly chosen for each mouse and 100–200 fibers per picture were calculated. The data are shown as percent of myelinated fibers and axonal diameter.

### RNA extraction and quantitative real-time PCR

Mice were euthanized, and tissue from the corpus callosum was collected for RNA extraction as previously reported [24]. Briefly, gross coronal sections were obtained between approximately Bregma −0.25 and −1.25 mm. Sagittal cuts were made through the cingulum, medial to each lateral ventricle, followed by cuts above and below the corpus callosum to remove most of the cortex and fornix [24]. Corpus callosum tissue samples were stored at −80 °C until required for further processing. Samples of fresh frozen corpus callosum were processed for RNA extraction using ISOGEN (Nippon Gene Co.) following the manufacturer’s instructions. Extracted RNA was resuspended in RNase-free molecular grade water (Takara Bio Inc.) and stored at −80 °C until required for analysis. For qPCR, total RNA (3 μg) was reverse transcribed using a PrimeScript RT reagent kit (Takara Bio Inc.). qPCR was performed using the 7900 Sequence Detection System (Applied Biosystems), with the following gene-specific primers: phosphoglycerate kinase 1 (PGK1, forward: 5′-ctgctgttccaagcatcaaa-3′ reverse: 5′-gcatcttttcccttcccttc-3′), glial fibrillary acidic protein (GFAP, forward: 5′-acgcttctccttgtctcgaa-3′ reverse: 5′-cggcgatagtcgttagcttc-3′), ionized calcium binding adapter molecule 1 (Iba1, forward: 5′-atgagccaaagcagggattt-3′ reverse: 5′-gaccagttggcctcttgtgt-3′), NOD-like receptor family, pyrin domain containing 3 (NLRP3, forward: 5′-ccttggaccaggttcagtgt-3′ reverse: 5′-aggagatgtcgaagcagcat-3′), purinergic receptor P2X ligand-gated ion channel 7 (P2X7R, forward: 5′-tgtgtgcattgacttgctca-3′ reverse: 5′-cttgcagacttttcccaagc-3′) Interleukin-1 beta (IL-1β, forward: 5′-gaccttccaggatgaggaca-3′ reverse: 5′-aggccacaggtattttgtcg-3′). Q-PCR conditions were 95 °C for 30 s, followed by 40 cycles of 5 s at 95 °C and 34 s at 60 °C. The level of target gene expression was calculated using the ΔΔC_T_ method [25]. Data were analyzed using the relative quantification technique. qPCR results were normalized to the expression levels of PGK1, as previously reported [21]. Relative changes in gene expression are reported as a percentage of the level of expression in control mice.

### Rotarod test

We used an accelerating rotarod treadmill for mice (Mouse Rotarod, UgoBasile) to evaluate motor balance and coordination following cuprizone exposure. Mice exposed to cuprizone for 5 weeks (2ccPA administration protocols A and B) were tested on the rotarod at 28 rpm, while those exposed to cuprizone for 10 weeks (2ccPA administration protocol D) were tested at 20 rpm. The time each mouse stayed on the rod (latency time) was recorded by a trip switch under the floor of each rotating drum, with a maximum recording time of 300 s. The number of falls (from the cylinder) and flips (when the animal clung to the cylinder) were also counted.

### Induction of EAE and 2ccPA treatment

Female C57BL/6J mice were immunized with MOG_35−55_/CFA emulsion pertussis toxin kits (EK-2110, Hooke laboratories) according to the manufacturer’s instructions [26, 27]. Briefly, 0.1 ml MOG_35−55_/CFA emulsion was injected subcutaneously into both flanks of each mouse (0.2 ml/animal, 200 μg of MOG_35−55_ peptide in each 0.2 ml dose). Mice then received intraperitoneal injections of pertussis toxin (0.1 ml/animal/day, 400 ng pertussis toxin in each 0.1 ml dose) on the same day and 24 h later. The day after the last injection of MOG was considered day 1. Clinical signs were scored as follows: 0, no clinical sign; 0.5, partial tail paralysis; 1.0, complete tail paralysis; 1.5, complete tail paralysis and discrete hind limb weakness; 2.0, complete tail paralysis and strong hind limb weakness; 2.5, unilateral hind limb paralysis; 3, complete hind limb paralysis; 3.5, hind limb paralysis and forelimb weakness; 4.0, complete paralysis (tetraplegia), and 5.0, moribund or dead [26, 27]. In each mouse, 2ccPA was administered at a dose of 16 mg/kg via intraperitoneal injection once daily for the duration of the EAE protocol (days 0–30 or 17–30). Sections were stained H&E. Five random sections from each mouse were observed to evaluate the degree of inflammation [28]. For evaluation of inflammation, a four-point scale was graded as follows: 0, no sign or minimal inflammation; 1, inflammatory cell infiltrates in meninges; 2, perivascular inflammatory cell infiltrates; and 3, marked infiltration of inflammatory cells into the parenchyma. The histological score represented the mean of the scores of all sections examined [28].

### Immunohistochemistry

Brain sections were incubated with rabbit anti-Iba1 antibody (Wako, 1:250), rabbit anti-CD4 antibody (Bioss, 1:250), and rat anti-F4/80 antibody (Bio-Rad, 1:250) at 4 °C overnight, followed by incubation at room temperature for 1 h with the secondary antibody (Cy3-conjugated AffiniPure goat anti-rabbit IgG, Jackson ImmunoReseach, 1:500). Sections were acquired using a KEYENCE BZ-X700 microscope (Keyence Corporation). The images were acquired sequentially using the 561 nm wavelength of a light-emitting diode (LED) to Cy3. All images were acquired using a UPLSAPO × 40 numerical aperture 0.95 dry objective lens (Olympus). The fluorescence intensity was measured by ImageJ 1.46r.

### Statistical analysis

The number of falls and flips was analyzed using a nonparametric Kruskal-Wallis test. EAE score was analyzed using a nonparametric Mann-Whitney *U* test. The protein levels of oligodendrocyte and astrocyte markers were analyzed using a student’s *t* test. All other data were analyzed by one-way analysis of variance (ANOVA) followed by Newman-Keuls post hoc test. All data were analyzed using GraphPad Prism Ver. 5.01 (Graphpad Software Inc.) and expressed as the mean ± SEM. *P* values <0.05 were considered statistically significant.

## Results

### 2ccPA protected oligodendrocyte cells from mitochondrial apoptosis

We undertook in vitro investigations using the MO3.13 oligodendrocyte cell line, an immortalized human-human hybrid cell line that can undergo differentiation into an oligodendrocyte phenotype. In the present study, undifferentiated MO3.13 cells with few processes (Fig. 2b) were cultured in DMEM without FBS for 5 days to induce differentiation. Serum-starved differentiated MO3.13 cells exhibited increases in process length (Fig. 2c). Differentiated cells exhibited expression of the oligodendrocyte markers MBP and Olig1 (Fig. 2d–f) and decreased expression of the astrocyte marker GS (Fig. 2d, g). No GFAP (another astrocytic marker) expression was detected in either phenotype (Fig. 2d). Differentiated MO3.13 cells were then used for subsequent experiments. To induce mitochondrial apoptosis in vitro, CoCl_2_ (cobalt chloride), a chemical mitochondrial apoptosis-inducing agent, was added in the presence or absence of 2ccPA, and cell viability was evaluated using MTT. Treatment with 2ccPA significantly increased cell viability in the presence of CoCl_2_ (Fig. 2h). The Bcl-2 family is an important regulator of mitochondrial dysfunction, which is induced via apoptosis pathways. Mitochondrial dysfunction induces an increase in the expression of proapoptotic proteins, such as Bax, and a decrease in the expression of antiapoptotic proteins, such as Bcl-2. Bax and Bcl-2 protein levels were determined by Western blot analysis (Fig. 2i), and the Bax/Bcl-2 protein expression ratio was evaluated (Fig. 2j). The Bax/Bcl-2 ratio increased with CoCl_2_ exposure. Treatment with 2ccPA suppressed the CoCl_2_-induced increase in the Bax/Bcl-2 ratio, which suggested that 2ccPA protected oligodendrocyte cells from CoCl_2_-induced mitochondrial apoptosis. In addition, we observed activation of p38MAPK and JNK during CoCl_2_-induced apoptosis [29, 30]. CoCl_2_ increased the phosphorylation levels of p38MAPK and JNK protein, which were suppressed by 2ccPA treatment (Fig. 2k, l). These data indicate that 2ccPA suppressed the CoCl_2_-induced apoptosis by inhibiting the phosphorylation of p38MAPK and JNK.

### 2ccPA suppressed the cuprizone-induced acute peak demyelination

**Fig. 3:**
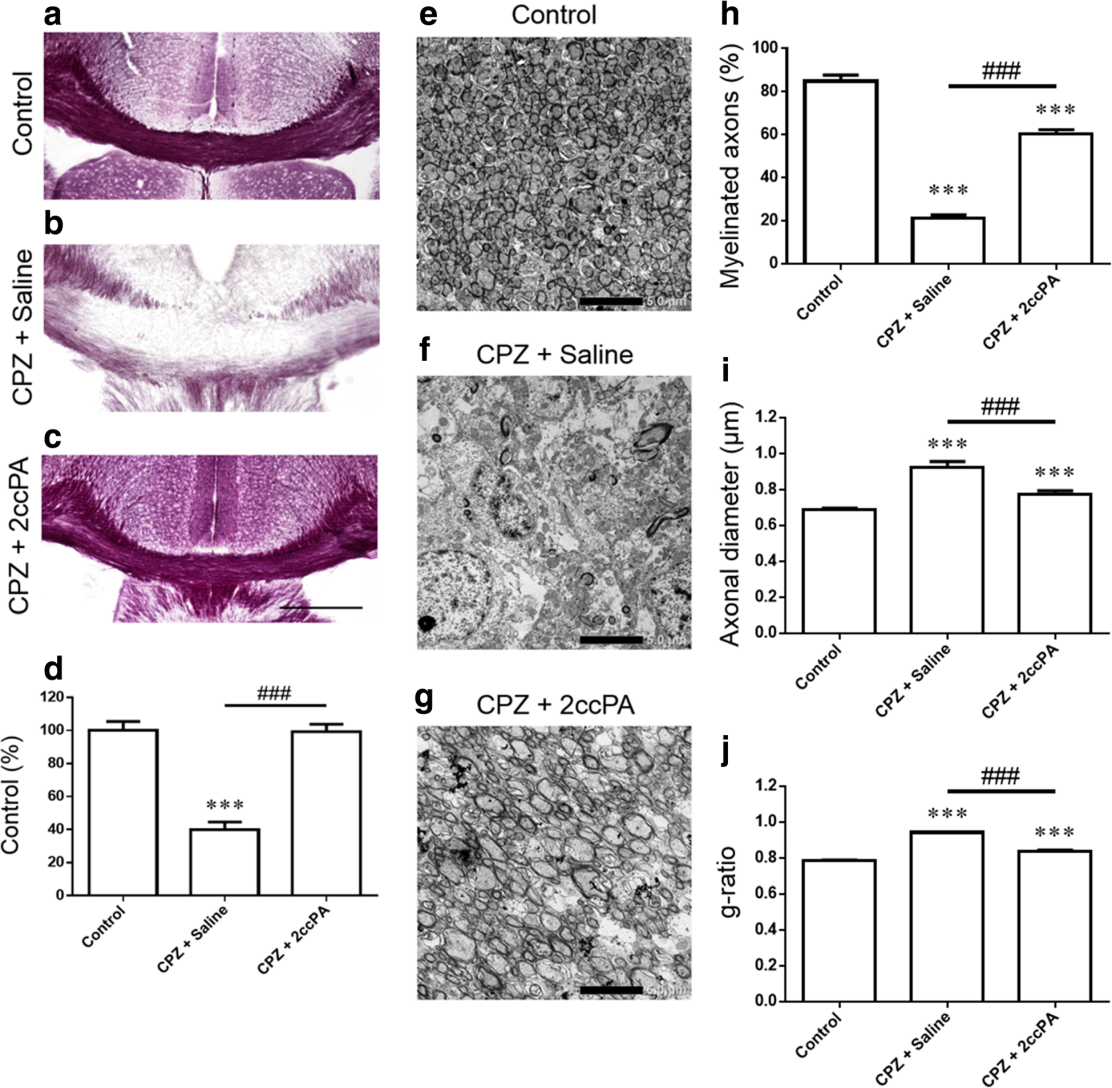
2ccPA suppressed the cuprizone-induced acute peak demyelination. Representative photomicrographs of coronal brain sections at the level of the fimbria demonstrate progressive demyelination of the corpus callosum after 5 weeks of cuprizone (CPZ) exposure. Black-Gold II staining in the control (**a**), CPZ + Ssaline (**b**), and CPZ + 2ccPA (**c**) groups. Myelin densities in the corpus callosum (**d**) were compared with those of controls and expressed as a percentage of the control value using the ImageJ analysis program. Data are mean ± SEM, *n* = 5 animals. *Scale bars* = 500 mm. Electron micrographs demonstrate an acute peak demyelination of the corpus callosum after 5 weeks of CPZ exposure. Electron micrographs of control (**e**), CPZ + Saline (**f**), and CPZ + 2ccPA (**g**) in the corpus callosum. Myelinated axons (**h**), axonal diameter (**i**), and g-ratio (**j**). Data are mean ± SEM, *n* = 7 animals. Statistical analysis was performed using one-way ANOVA followed by post hoc Newman-Keuls test (***p < 0.001 vs. control; ^###^ *p* < 0.001 vs. CPZ + Saline). *Scale bar* = 5 μm

To investigate the effects of 2ccPA on cuprizone-induced acute peak demyelination, we fed mice a cuprizone diet and administrated 2ccPA for 5 weeks (Fig. 1, protocol A). Myelin content was quantified using Black-Gold II staining. In control mice, the corpus callosum appeared to retain sufficient myelin content (Fig. 3a). Five weeks of cuprizone exposure induced acute peak demyelination in the corpus callosum (Fig. 3b). 2ccPA treatment suppressed the acute peak demyelination almost completely (Fig. 3c, d). Electron microscopy was then used to obtain data for quantitative analysis of myelinated axons, axonal diameter, and g-ratios. Control mice exhibited full myelination in the corpus callosum (Fig. 3e). In contrast, mice exposed to cuprizone for 5 weeks exhibited a decrease in the extent of myelination and residual myelin sheaths (Fig. 3f), while treatment with 2ccPA attenuated this decrease in myelination (Fig. 3g). In addition, 2ccPA treatment significantly increased the number of myelinated axons (Fig. 3h), reduced axonal diameter (Fig. 3i), and lowered g-ratios (Fig. 3j) in comparison to values obtained for cuprizone-treated mice. These findings indicate that treatment with 2ccPA suppresses cuprizone-induced axonal damage and demyelination.

### 2ccPA suppressed the neuroinflammation

**Fig. 4:**
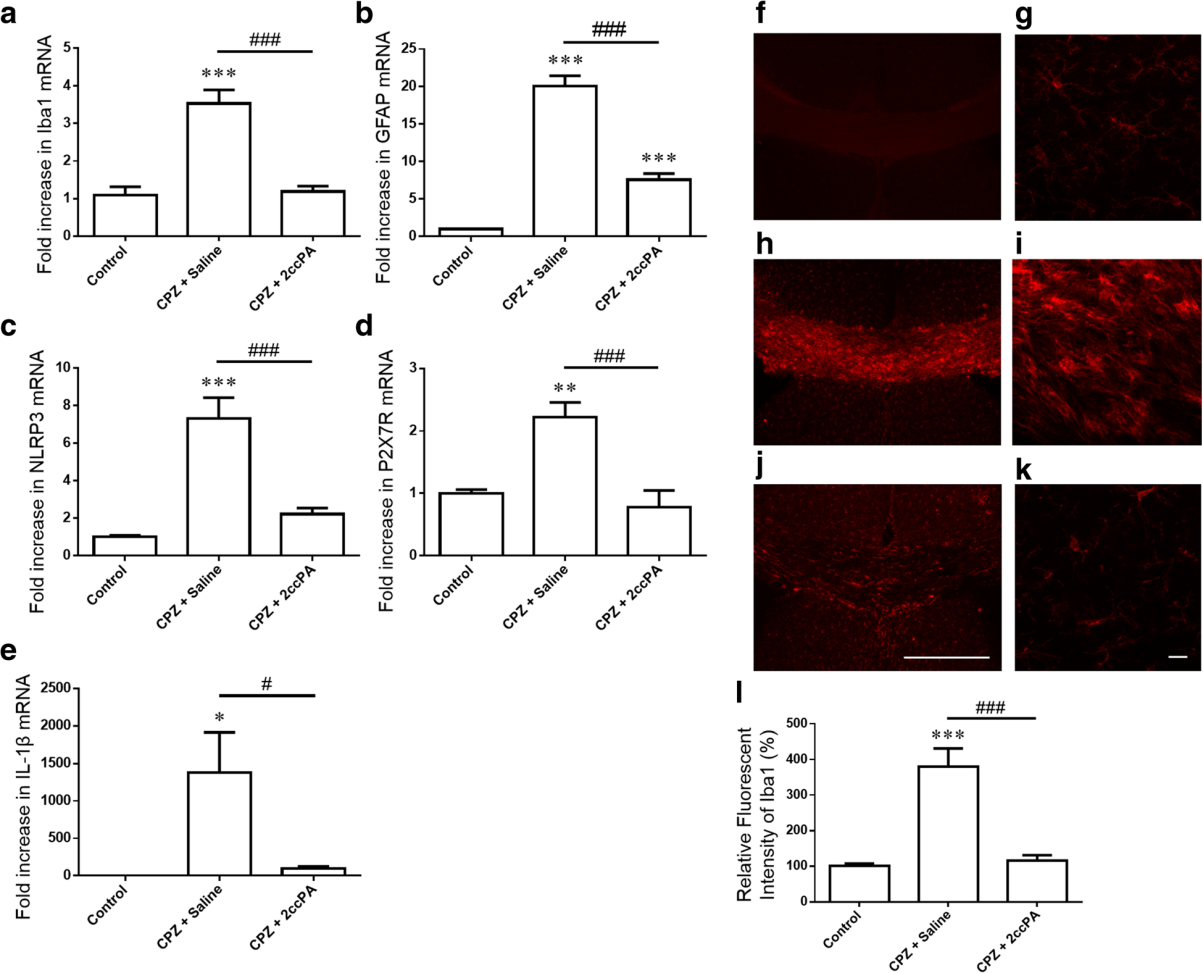
2ccPA suppressed the neuroinflammation. The mRNA levels of Iba1 (**a**), GFAP (**b**), NLRP3 (**c**), P2X7R (**d**), and IL-1β (**e**) relative to PGK1, as determined by qPCR in the corpus callosum after 5 weeks of CPZ exposure. Data are mean ± SEM, *n* = 5 animals. Immunostaining with anti-Iba1 showed microglial activation in the corpus callosum. Control (**f**, **g**), CPZ + saline (**h**, **i**), and CPZ + 2ccPA (**j**, **k**). Number of microglia in the corpus callosum (**l**). Data are mean ± SEM, *n* = 6 animals. Statistical analysis was performed using one-way ANOVA followed by post hoc Newman-Keuls test (*p < 0.05; **p < 0.01; ***p < 0.001 vs. control; ^#^ *p* < 0.01; ^###^ *p* < 0.001 vs. CPZ + saline). *Scale bar* 500-μm left images; 50-μm right images

We evaluated glial activation and inflammasome formation to investigate the effect of 2ccPA on the neuroinflammation associated with demyelination. Levels of gene expression for the microglial marker (Iba1) and astrocytic marker (GFAP) were evaluated after 5 weeks of cuprizone. Cuprizone exposure increased messenger RNA (mRNA) levels of Iba1 and GFAP. 2ccPA treatment suppressed cuprizone-induced increases in Iba1 and GFAP mRNA expression (Fig. 4a, b). Previous studies have demonstrated that the NLRP3 inflammasome plays an essential role in neuroinflammatory diseases including multiple sclerosis [9]. We analyzed the gene expression levels of NLRP3 inflammasome-related genes such as NLRP3, P2X7R, and IL-1β. NLRP3, P2X7R, and IL-1β expression levels were increased by cuprizone exposure and were suppressed by 2ccPA treatment (Fig. 4c–e). Iba1-positive microglia were detected by immunofluorescence analysis in the corpus callosum (Fig. 4f–k). Microglia were seen only sporadically in the corpus callosum of the control mice (Fig. 4f, g). Mice exposed to cuprizone exhibited hypertrophic microglia with enlarged cell bodies (Fig. 4h, i), which were suppressed by 2ccPA treatment (Fig. 4j, k). The cuprizone-induced increases in microglia in the corpus callosum were reduced by 2ccPA treatment (Fig. 4l). We observed no significant difference in levels of gene expression for the alternative markers of microglial activation Arg1, Fizz1, and Ym1 among corpus callosum tissue samples (Control, CPZ + saline, CPZ + 2ccPA) (data not shown). These findings indicate that treatment with 2ccPA effectively suppressed cuprizone-induced NLRP3 inflammasome formation and microglial activation.

### Beneficial effects of 2ccPA in the multiperiod of cuprizone-induced demyelination

**Fig. 5:**
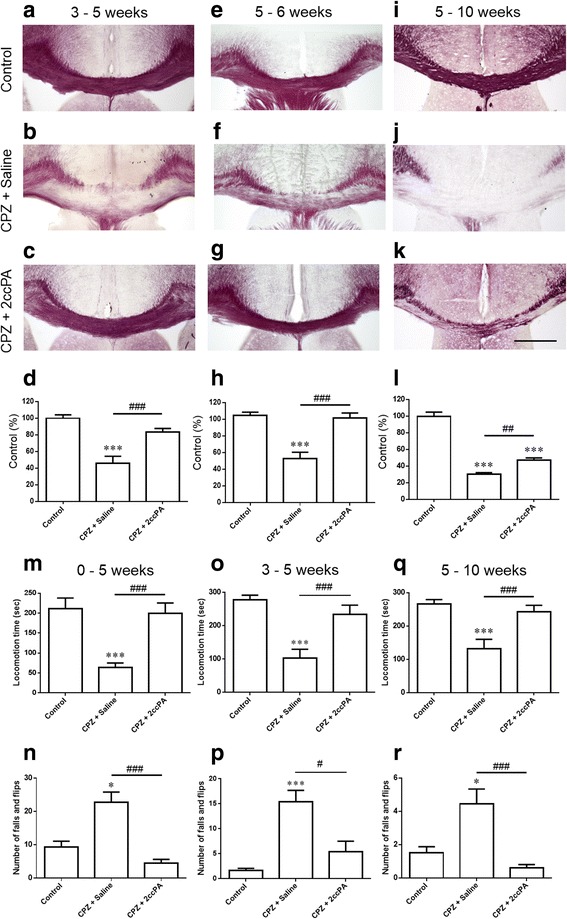
Effects of 2ccPA in the multiperiod of CPZ-induced demyelination and motor dysfunction. Black-Gold II staining of control (**a**, **e**, and **i**), CPZ + saline (**b**, **f**, and **j**), CPZ + 2ccPA (**c**, **g**, and **k**). Myelin densities in the corpus callosum (**d**, **h**, and **l**) were compared with controls and expressed as a percentage of control value using the ImageJ analysis program. Data are mean ± SEM., *n* = 5 animals. Statistical analysis was performed using one-way ANOVA followed by post hoc Newman-Keuls test (***p < 0.001 vs. control; ^##^ *p* < 0.01; ^###^ *p* < 0.001 vs. CPZ + saline). *Scale bars* = 500 μm. Motor performance on the rotarod. Mice were assessed for locomotion time during a period of 300 s (**m**, **o**, and **q**). Data are mean ± SEM., *n* = 12 animals. Statistical analysis was performed using one-way ANOVA followed by post hoc Newman-Keuls test (***p < 0.001 vs. control; ^###^ *p* < 0.001 vs. CPZ + saline). Number of falls and flips (**n**, **p**, and **r**). Data are mean ± SEM., *n* = 12 animals (0–5 weeks group), *n* = 10 animals (3–5 weeks group), and *n* = 15 animals (5–10 weeks group). Statistical analysis was performed using non-parametric Kruskal-Wallis test (*p < 0.05; ***p < 0.001 vs. control; ^#^ *p* < 0.05; ^###^ *p* < 0.001 vs. CPZ + saline)

We investigated the therapeutic potential of 2ccPA in cuprizone-induced demyelination. In three experimental settings, 2ccPA treatment was administrated during at weeks 3–5 (protocol B: beginning of substantial demyelination and motor dysfunction), weeks 5–6 (protocol C: spontaneous remyelination), or weeks 5–10 (protocol D: chronic severe demyelination) of cuprizone exposure. Treatment with 2ccPA (protocol B) suppressed acute peak demyelination after the onset of cuprizone-induced neurological symptoms (Fig. 5a–d). It is well known that spontaneous remyelination occurs following acute demyelination in the corpus callosum of cuprizone model mice. At 6 weeks of cuprizone exposure, we observed spontaneous remyelination in cuprizone mice (Fig. 5f). Treatment with 2ccPA during the final week (protocol C) significantly promoted spontaneous remyelination (Fig. 5e–h). Prolonged cuprizone exposure resulted in chronic demyelination. Treatment with 2ccPA (5–10 weeks) reduced chronic demyelination (Fig. 5i–l). These results demonstrate that 2ccPA exerts beneficial effects in the multiperiod of cuprizone-induced demyelination.

### 2ccPA improved cuprizone-induced motor dysfunction

To investigate the effects of 2ccPA on motor dysfunction caused by cuprizone-induced demyelination, we assessed the locomotor coordination and balance of mice using a rotarod apparatus. Mice exposed to cuprizone (0–5 and 5–10 weeks) exhibited significant decreases in locomotion time as well as significant increases in the number of falls and flips. Treatment with 2ccPA (protocols A, B, and D) significantly restored locomotion time remarkably (Fig. 5m, o, and q) and suppressed the number of falls and flips (Fig. 5n, p, and r). 2ccPA treatment significantly suppressed the cuprizone-induced impairment of motor performance.

### 2ccPA ameliorated EAE pathology

**Fig. 6:**
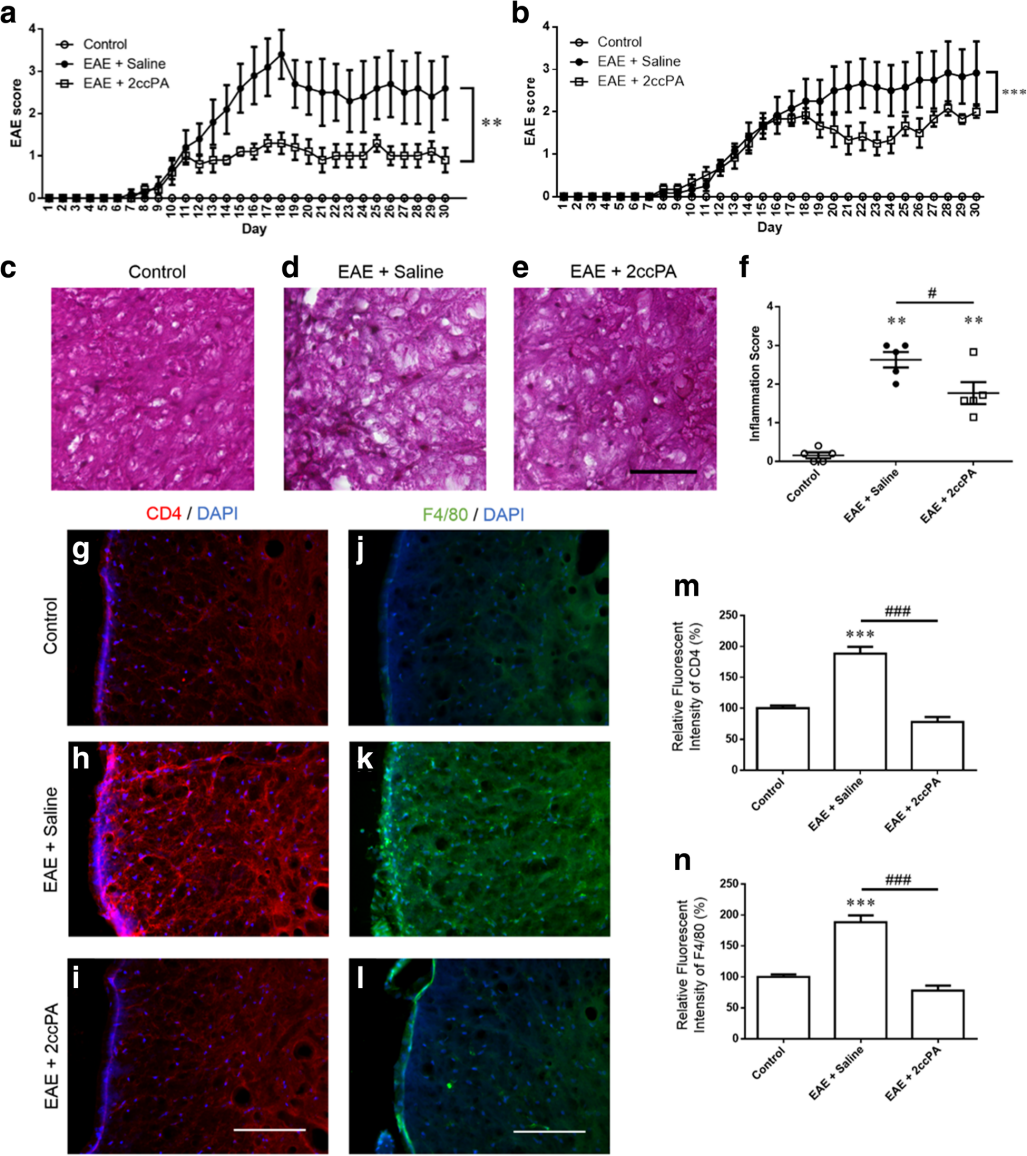
2ccPA-ameliorated EAE pathological condition and immunopathology. Mice treated with EAE + saline and EAE + 2ccPA were immunized with the MOG_35−55_ peptide and clinical scores were assessed daily for 30 days (**a**). Treatment with 2ccPA after the peak of EAE symptoms had occurred (**b**). Data are mean ± SEM, *n* = 5 animals. Statistical analysis was performed using two-way ANOVA followed by post hoc Mann-Whitney *U* test regarding day 0–30 (**a**) or day 17–30 (**b**), respectively, (**p < 0.01; ***p < 0.001 vs. EAE + saline). H&E staining revealed histological features of MOG_35−55_-induced EAE. Control (**c**), EAE + saline (**d**), EAE + 2ccPA (**e**). The degrees of inflammation (**f**). *Scale bar* = 50 μm. Immunostaining with T cell and macrophage infiltration in the spinal cord. Fluorescence for CD4, DAPI, and marge images. Control (**g**), EAE + saline (**h**), EAE + 2ccPA (**i**). Number of infiltrating T cells in the spinal cord (**m**). Fluorescence for F4/80, DAPI, and marge images. Control (**j**), EAE + saline (**k**), EAE + 2ccPA (**l**). Number of infiltrating macrophages in the spinal cord (**n**). Data are mean ± SEM, *n* = 5 animals. Statistical analysis was performed using one-way ANOVA followed by post hoc Newman-Keuls test (**p < 0.01; ***p < 0.001 vs. control; ^#^ *p* < 0.05; ^###^ *p* < 0.001 vs. EAE + saline). *Scale bar* = 100 μm

We assessed the potential of 2ccPA to improve the disease course in the EAE model mice. Mice were monitored daily for clinical symptoms and scored in accordance with established criteria [31]. Control mice exhibited no obvious symptoms of EAE disease. In contrast, EAE mice developed severe EAE symptoms, although treatment with 2ccPA significantly reduced clinical EAE scores. Indeed, a large difference in disease severity between the control and treatment groups was observed throughout the observation period (Fig. 6a). Mean clinical EAE scores were significantly lower for 2ccPA-treated mice than EAE mice. Although EAE mice peaked at scores of 3–4 following immunization, 2ccPA-treated mice peaked at scores of 1 following immunization. We then examined the therapeutic potential of 2ccPA in mice already exhibiting EAE symptoms. 2ccPA successfully attenuated clinical EAE symptoms even after the peak stages of disease (Fig. 6b), suggesting that treatment with 2ccPA dramatically suppresses impairments in neurological function. Inflammatory infiltration of immune cells in the spinal cord is a well-documented histological feature of the EAE model. Inflammatory infiltration of immune cells in the spinal cords was a histological feature of the EAE model. The spinal cords were stained with H&E to assess the degree of inflammation. Inflammatory cells penetrated the pia mater and infiltrated the perivascular regions and parenchyma. Control myelin of the white matter was highlighted clearly by H&E staining (Fig. 6c). In EAE mice, we found mononuclear infiltration in the leptomeninges and scattered throughout the white matter parenchyma (Fig. 6d). We observed severe inflammation with vacuolation in the anterior and lateral funiculi of the spinal cord. Treatment with 2ccPA reduced infiltration of mononuclear cells (Fig. 6e). Treatment with 2ccPA suppressed sustained scores for inflammation in the spinal cord (Fig. 6f).

### 2ccPA suppressed CD4- or F4/80-positive T cell and macrophage infiltration in the spinal cord

Infiltration of immune cells (T cells and macrophages) was observed in mice exposed to EAE, although these increases were suppressed following 2ccPA administration. Immunofluorescence in the spinal cord was evaluated using the T cell marker CD4 and macrophage marker F4/80. In control mice, CD4- and F4/80-positive cells were sporadically distributed in the spinal cord (Fig. 6g, j), while an accumulation of infiltrative cells was observed in meningeal area of the spinal cord in mice exposed to EAE (Fig. 6h, k). Mice exposed to EAE exhibited T cell and macrophage infiltration, although such infiltration was suppressed by 2ccPA treatment (Fig. 6i, l). Therefore, treatment with 2ccPA effectively suppressed EAE-induced T cell and macrophage infiltration in the spinal cord (Fig. 6m, n).

## Discussion

Multiple sclerosis is a heterogeneous disease in clinical presentation, in terms of demyelinating lesions, immunopathological subtypes, response to therapy, and genetic associations [6, 32]. A detailed immunopathological investigation of demyelinating lesions revealed four distinct immunopathological patterns [6]. In patterns I and II, demyelination occurs as a consequence of an autoimmune reaction against myelin, whereas demyelination is independent of immune activation and is caused by oligodendrocyte primary cell loss in patterns III and IV. Previous studies have revealed that administration of EAE and cuprizone reproduces the pathology observed in patterns I/II and patterns III/IV, respectively [33]. These animal models accurately represent all aspects of the pathology and clinical features of human multiple sclerosis [34]. The findings of the present study demonstrate that treatment with 2ccPA improved cuprizone-induced motor dysfunction and pathological EAE scores, suggesting that 2ccPA may exert beneficial effects in all subtypes of human multiple sclerosis.

Neuroinflammation (e.g., lymphocyte/macrophage infiltration, microglial activation, enhanced cytokine/chemokine production, demyelination, and axonal damage [35–37]) is a key component of the pathological progression of all subtypes of multiple sclerosis and in both the EAE and cuprizone models. In the current study, we demonstrated that 2ccPA suppressed neuroinflammation in the EAE and cuprizone models. 2ccPA treatment suppressed the infiltration of CD4-positive T cells and F4/80-positive macrophages to the spinal cord in EAE model mice. CD4-positive T cells secrete proinflammatory cytokines, which play important roles in the neuroinflammatory, cascade, and mediate the damage to the myelin sheath, demyelination, and eventually damage to the neuronal axon [38, 39]. F4/80-positive microglia/macrophages produce and respond to a wide variety of cytokines, impair blood-brain barrier function, act as antigen-presenting cells within the CNS, mediate phagocytic events, and damage oligodendrocytes [40, 41]. Therefore, our data suggest that inhibition of T cells and macrophage infiltrates into CNS by 2ccPA treatment should be associated with suppression of neuroinflammation in EAE. Further, we also demonstrated that 2ccPA suppressed cuprizone-induced microglial activation and NLRP3 inflammasome formation. Previous studies have reported that demyelination occurs in parallel with microglial activation in the cuprizone model [9]. Activated microglia contribute to the death of oligodendrocytes by secreting proinflammatory cytokines [42] and to axonal damage [43] by stripping synaptic proteins [44]. The NLRP3 inflammasome signaling pathway is involved in various neuroinflammatory diseases, including multiple sclerosis and the EAE and cuprizone models. Activation of the P2X7 receptor, principally by extracellular ATP, induces NLRP3 inflammasome activation and promotes the processing and release of IL-1β. IL-1β is a pivotal mediator in the neuroinflammatory response [9] and promotes leukocyte infiltration by inducing the expression of many cytokines, chemokines, and adhesion molecules. A recent study has reported that microglia express the NLRP3 inflammasome and release IL-1β [45], suggesting that the microglial NLRP3 inflammasome probably promotes CNS inflammation and demyelination. In our study, 2ccPA suppressed cuprizone-induced microglial activation and NLRP3 inflammasome, suggesting that 2ccPA suppressed the neuroinflammation to inhibit the microglial NLRP3 inflammasome caused by excessive microglial activation. Microglia are known to develop diverse functional phenotypes of proinflammatory (M1) and alternative (M2) activation [46, 47]. Previous findings have revealed that NLRP3 promotes microglial M1 activation and that the NLRP3 complex is contained in M1 microglia [48]. In the present study, 2ccPA treatment suppressed markers of the NLRP3 inflammasome (NLRP3, P2X7, and IL-1b) but not of M2 microglia (Arg1, Fizz1, and Ym1), suggested that 2ccPA suppressed the microglial M1 proinflammatory activation. Taken together, these findings suggest that 2ccPA improves the pathological state of EAE and cuprizone model mice by mediating the attenuation of neuroinflammatory conditions (i.e., infiltration of immune cells and microglial NLRP3 inflammasome, respectively).

Under neuroinflammatory conditions, mitochondrial apoptotic cell death has been observed following damage to the CNS [49]. Mitochondrial dysfunction plays a crucial role in the loss of oligodendrocytes and neuronal axons in multiple sclerosis [50] and in the EAE and cuprizone models. The mitochondria-mediated apoptosis pathway is largely controlled by the master apoptosis inducer Bax and the apoptosis suppressor Bcl-2 [51]. Phosphorylation and activation of JNK and p38MAPK also promote mitochondrial apoptotic cell death [52, 53]. In this study, we showed that 2ccPA suppressed CoCl_2_-induced apoptosis. We reported previously that natural cPA suppressed mitochondrial apoptosis of neuronal cells in vitro and delayed neuronal death in vivo. Therefore, our results suggest that the protective function of 2ccPA suppressed the mitochondrial apoptosis pathway in both oligodendrocytes and neurons, which is likely to be associated with protection from the loss of oligodendrocytes and axons in demyelinative conditions.

Previously, we reported that 2ccPA promotes neurite outgrowth and enhances neuronal survival via a signaling pathway similar to that of NGF [11]. Therefore, it is possible that 2ccPA exerts protective effects against cuprizone-induced demyelination and EAE pathology via NGF-like actions. Previous studies have indicated that NGF exerts a dramatic effect on neuron and oligodendrocyte survival and stimulates axonal regeneration/remyelination [54, 55]. These findings are in accordance with the protective effects against CPZ-induced demyelination observed in our study. Additional studies have indicated that NGF may exert protective effects in EAE model mice by switching the immune response to an anti-inflammatory status [56, 57]. Indeed, our findings indicate that a similar process may underlie the protective effect of 2ccPA against EAE. Furthermore, NGF is a potent anti-apoptotic factor that regulates levels of anti-apoptotic Bcl-2 protein, a common mechanism by which 2ccPA protected oligodendrocytes from CoCl_2_-induced apoptosis [58]. These findings suggest that multiple effects of 2ccPA are due to its NGF-like actions.

There is a need for therapeutic drug treatment of progressive multiple sclerosis that can arrest the progression of demyelination. Our results may indicate that 2ccPA has a beneficial effect on progressive demyelination. Treatment with 2ccPA suppressed demyelination and motor dysfunction even after the onset of cuprizone-induced pathology (protocol B and D). Further, 2ccPA might have a function to enhance remyelination (protocol C). In summary, we found that the administration of 2ccPA reduced cuprizone-induced demyelination, microglial activation, NLRP3 inflammasome, and motor dysfunction, and promoted remyelination. Furthermore, we found that 2ccPA reduced autoreactive T cell and macrophage, spinal cord injury, and clinical behavioral dysfunction in the autoimmune multiple sclerosis model of EAE. These data indicate that 2ccPA may be a promising seed compound for the development of new drugs to treat demyelinating disease and ameliorate the symptoms of multiple sclerosis.

## Conclusions

The findings of the present study demonstrate that 2ccPA protected oligodendrocytes via suppression of the mitochondrial apoptosis pathway. Beneficial effects of 2ccPA were observed in the multiperiod of cuprizone-induced demyelination and EAE pathology. These data indicate that 2ccPA is a promising candidate for the development of new drugs for the treatment of demyelinating conditions such as multiple sclerosis.

## Abbreviations

2ccPA: 2-Carba-cyclic phosphatidic acid
cPA: Cyclic phosphatidic acid
CPZ: Cuprizone
EAE: Experimental autoimmune encephalomyelitis
GFAP: Glial fibrillary acidic protein
Iba1: Ionized calcium binding adapter molecule 1
IL-1β: Interleukin-1 beta
NLRP3: NOD-like receptor family, pyrin domain containing 3
P2X7: Purinergic receptor P2X ligand-gated ion channel 7
