# Correction to: Protective and therapeutic role of 2-carbacyclic phosphatidic acid in demyelinating disease

**DOI:** 10.1186/s12974-018-1104-x

**Published:** 2018-03-05

**Authors:** Shinji Yamamoto, Kota Yamashina, Masaki Ishikawa, Mari Gotoh, Sosuke Yagishita, Kensuke Iwasa, Kei Maruyama, Kimiko Murakami-Murofushi, Keisuke Yoshikawa

**Affiliations:** 1 Department of Pharmacology, Faculty of Medicine, Saitama Medical University, 38 Moro-hongo, Moroyama-machi, Iruma-gun, Saitama, 350-0495 Japan; 2 Endowed Research Division of Human Welfare Sciences, Ochanomizu University, 2-1-1 Ohtsuka, Bunkyo-ku, Tokyo, 112-8610 Japan

## Correction

After publication of the article [1], it was brought to our attention that an acknowledgement was missing from the original version. The authors would also like to include “We appreciate that Dr. Yoshibumi Shimizu at Ochanomizu University for sharing with us his preliminary experimental results about 2ccPA in the mouse brain following intraperitoneal administration”.

## References

[1] Yamamoto S, Yamashina K, Ishikawa M, Gotoh M, Yagishita S, Iwasa K, et al. Protective and therapeutic role of 2-carba-cyclic phosphatidic acid in demyelinating disease. J Neuroinflammation. 2017;14(1) 10.1186/s12974-017-0923-5. 10.1186/s12974-017-0923-5 PMC5521126 28732510

